# Prognostic value of MET protein overexpression and gene amplification in locoregionally advanced nasopharyngeal carcinoma

**DOI:** 10.18632/oncotarget.3751

**Published:** 2015-04-20

**Authors:** Yingqin Li, Wenfei Li, Qingmei He, Yafei Xu, Xianyue Ren, Xinran Tang, Xin Wen, Xiaojing Yang, Ying Sun, Jing Zeng, Jingping Yun, Na Liu, Jun Ma

**Affiliations:** ^1^ Sun Yat-sen University Cancer Center, State Key Laboratory of Oncology in South China, Collaborative Innovation Center of Cancer Medicine, Guangzhou, China; ^2^ Department of Pathology, State Key Laboratory of Oncology in South China, Sun Yat-sen University Cancer Center, Guangzhou, China

**Keywords:** MET overexpression, MET amplification, prognosis, locoregionally advanced nasopharyngeal carcinoma

## Abstract

This study assessed the incidence and prognostic value of MET protein overexpression and gene amplification in locoregionally advanced nasopharyngeal carcinoma (NPC). Specimens from 376 consecutive patients with locoregionally advanced NPC were subjected to immunohistochemistry to analyze MET protein expression and fluorescence *in situ* hybridization to assess *MET* amplification status. In total, 139/376 (37.0%) patients had MET protein overexpression; of whom, 7/139 (5.0%) had *MET* amplification. MET overexpression was significantly associated with locoregional failure (*P* = 0.009), distant metastasis (*P* = 0.006) and death (*P* < 0.001); *MET* amplification was significantly associated with death (*P* = 0.021). A positive correlation was observed between *MET* copy number status and MET protein expression (*r* = 0.629, *P* < 0.001). Multivariate analysis demonstrated MET overexpression was an independent prognostic factor for overall survival (OS; HR, 1.99; 95% CI, 1.38–2.87; *P* < 0.001) and disease-free survival (DFS; HR, 1.85; 95% CI, 1.33–2.57; *P* < 0.001), and *MET* amplification was independently associated with poorer OS (HR, 4.24; 95% CI, 1.78-10.08; *P* < 0.001) and DFS (HR, 5.44; 95% CI, 2.44-12.09; *P* < 0.001). In conclusion, MET protein overexpression and gene amplification are independent prognostic factors for OS and DFS in locoregionally advanced nasopharyngeal carcinoma, and may provide therapeutic biomarkers to identify patients in whom MET inhibitors may be beneficial.

## INTRODUCTION

Nasopharyngeal carcinoma (NPC) is one of the most common head and neck malignancies in Southern China, where the rates vary from 30 to 80 cases per 100,000 [1, 2]. Unfortunately, about 80% of patients with NPC are diagnosed with advanced disease at their first visit, due to the deep anatomical position and non-specific symptoms of this tumor type [3]. Intensity-modulated radiotherapy combined with platinum-based chemoradiotherapy is the standard treatment for locoregionally advanced NPC [4, 5]. Although local and regional control rates have improved, more than 30% of patients still develop recurrence and distant metastasis [6, 7]. These challenges urgently require the development of novel therapeutic strategies to improve the clinical outcome of patients with locoregionally advanced NPC. Recently, a new generation of molecular targeted drugs has been added to traditional chemotherapy and demonstrated therapeutic efficacy [8, 9]. Therefore, identification of novel molecular aberrations in specific subgroups of patients may facilitate the development of other targeted therapies and guide individualized treatment protocols for patients with NPC.

The *MET* proto-oncogene encodes the receptor tyrosine kinase MET, which is activated by its ligand, hepatocyte growth factor (HGF) [10, 11]. Binding of HGF to MET results in phosphorylation of the receptor tyrosine kinase domain, and in turn mediates downstream signaling via the PI3K/AKT, STAT3, RAS-RAC/RHO and MAPK pathways [12, 13]. Normal MET signaling is required for embryogenesis, cell growth, cell differentiation and angiogenesis [14, 15]. Constitutive activation of the MET pathway has been reported in various types of cancer, and promotes tumor cell proliferation, motility, invasion and metastasis [16, 17]. Aberrant MET activation can occur via multiple mechanisms, including gene amplification and mutation, dysregulation of microRNAs that target *MET*, paracrine or autocrine activation via HGF, and protein overexpression [18, 19]. Recent studies demonstrated MET overexpression and *MET* amplification were associated with poorer clinical outcomes in non-small cell lung cancer and gastric cancer [20–22]. On this basis, MET has emerged as a potential target for anticancer therapy.

Currently, a number of MET inhibitors have been developed and are in clinical trials at different phases [23–26]. The preliminary results of these trials have demonstrated MET inhibitors have variable benefits in unselected patient cohorts, which suggests the need for a selective biomarker to indicate subgroups of patients that may potentially obtain more benefit from MET inhibitors. Preclinical studies indicated *MET* amplification can be used to identify subgroups of patients with gastric cancer who are sensitive to MET inhibitors [27]. However, the frequency and prognostic value of MET overexpression in NPC remains controversial due to the use of varied detection methods, cutoff criteria and populations in different studies [28–30]. More importantly, the frequency of *MET* amplification in NPC has not yet been determined.

Therefore, in this study, we aimed to evaluate the frequency of MET protein overexpression in patients with locoregionally advanced NPC, and the incidence of *MET* amplification in patients overexpressing MET. In addition, we analyzed the association with clinicopathological features, as well as the prognostic value of MET overexpression and *MET* amplification to assess the value of MET as a potential therapeutic target for personalized treatment of patients with NPC.

## RESULTS

### MET protein expression in NPC

**Figure 1:**
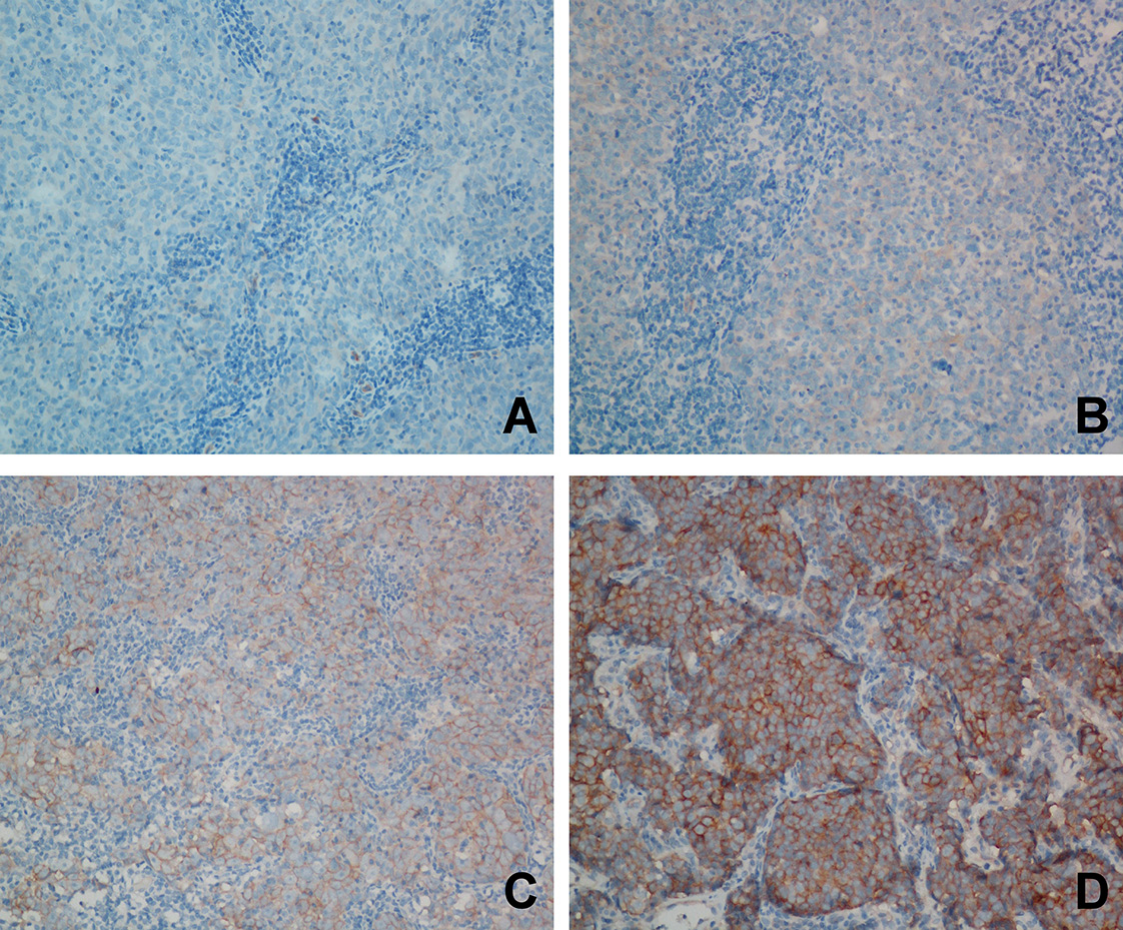
Representative images of immunohistochemical (IHC) staining for MET in locoregionally advanced nasopharyngeal carcinoma **A.** MET IHC score of 0. **B.** MET IHC score of 1+. B. MET IHC score of 2+. **C.** MET IHC score of 3+.

MET protein expression was evaluated in 376 patients with locoregionally advanced nasopharyngeal carcinoma. Overall, the tumors of 74 (19.7%), 163 (43.3%), 104 (27.7%) and 35 (9.3%) patients had a MET immunohistochemical (IHC) staining score of 0, 1+, 2+ and 3+, respectively (Figure 1A–1D). Overexpression of MET was observed in 37.0% (139/376) of the NPC tissues. The groups of patients with high MET expression and low MET expression had similar distributions of host and tumor factors. In addition, there was no significant difference with regards to the radiotherapy (RT) technique or use of chemotherapy between groups. However, a lower frequency of WHO type IIb NPC was observed in patients with high MET expression compared to patients with low MET expression (90.6% vs. 97.5%, *P* = 0.004; Table 1). Moreover, patients with high MET expression had a higher incidence of locoregional failure (25.2% vs. 14.3%, *P* = 0.009), distant metastasis (28.8% vs. 16.9%, *P* = 0.006) and death (41.7% vs. 23.6%, *P* < 0.001; Table 1).

**Table 1:** Association of MET protein expression and *MET* amplification status with the clinical characteristics of patients with locoregionally advanced nasopharyngeal carcinoma

Characteristic	MET expression (*n* = 376)		*P*	MET amplification (*n* = 139)		*P*
	Low *n* (%), (*n* = 237)	High *n* (%), (*n* = 139)		Negative *n* (%), (*n* = 132)	Positive *n* (%), (*n* = 7)	
**Age**						
≤ 45 years	120 (50.6)	67 (48.2)	0.694	64 (48.5)	3 (42.9)	1.000
> 45 years	117 (49.4)	72 (51.8)		68 (51.5)	4 (51.1)	
**Gender**						
Male	175 (73.8)	107 (77.0)	0.497	100 (75.8)	7(100.0)	0.352
Female	62 (26.2)	32 (23.0)		32 (24.2)	0 (0.0)	
**WHO Type**						
IIa	6 (2.5)	13 (9.4)	**0.004**	13 (9.8)	0 (0)	1.000
IIb	231 (97.5)	126 (90.6)		119 (90.2)	7 (100)	
**VCA–IgA**						
< 1:80	33 (13.9)	19 (13.7)	0.945	18 (13.6)	1 (14.3)	0.107
≥ 1:80	204 (86.1)	120 (86.3)		114 (86.4)	6 (85.7)	
**EA-IgA**						
< 1:10	50 (21.1)	26 (18.7)	0.577	26 (19.7)	0 (0)	0.348
≥ 1:10	187 (78.9)	113 (81.3)		106 (80.3)	7 (100)	
**T Stage**						
T1–T2	40 (16.9)	19 (13.7)	0.409	19 (14.4)	0 (0)	0.593
T3–T4	197 (83.1)	120 (86.3)		113 (85.6)	7 (100)	
**N Stage**						
N0–N1	125 (52.7)	84 (60.4)	0.147	80 (60.6)	4 (57.1)	1.000
N2–N3	112 (47.3)	55 (39.6)		52 (39.4)	3 (42.9)	
**TNM Stage**						
III	134 (56.5)	79 (56.8)	0.956	75 (56.8)	4 (57.1)	1.000
IV	103 (43.5)	60 (43.2)		57 (43.2)	3 (42.9)	
**Chemoradiotherapy**						
Yes	197 (83.1)	114 (82.0)	0.784	107 (81.1)	7 (100)	0.351
No	40 (16.9)	25 (18.0)		25 (18.9)	0 (0)	
**Radiotherapy**						
IMRT	29 (12.2)	10 (7.2)	0.122	10 (7.6)	0 (0)	1.000
2D–RT	208 (87.8)	129 (92.8)		122 (92.4)	7 (100)	
**Locoregional failure**						
Yes	34 (14.3)	35 (25.2)	**0.009**	33 (25.0)	2 (28.6)	1.000
No	203 (83.1)	104 (74.8)		99 (75.0)	5 (71.4)	
**Distant metastasis**						
Yes	40 (16.9)	40 (28.8)	**0.006**	37 (28.0)	3 (42.9)	0.410
No	197 (85.6)	99 (71.2)		95 (72.0)	4 (57.1)	
**Death**						
Yes	56 (23.6)	58 (41.7)	**< 0.001**	52 (39.4)	6 (85.7)	**0.021**
No	181 (76.4)	81 (58.3)		80 (60.6)	1 (14.3)	

Abbreviations: WHO type IIa, differentiated non-keratinizing nasopharyngeal carcinoma; WHO type IIb, undifferentiated non-keratinizing nasopharyngeal carcinoma; VCA–IgA, viral capsid antigen immunoglobulin A; EA–IgA, early antigen immunoglobulin A; IMRT: intensity-modulated radiotherapy; 2D–RT: two-dimensional radiotherapy. All patients were restaged according to the 7^th^ edition of the AJCC Cancer Staging Manual.

### *MET* copy number status in patients with high MET expression

**Figure 2:**
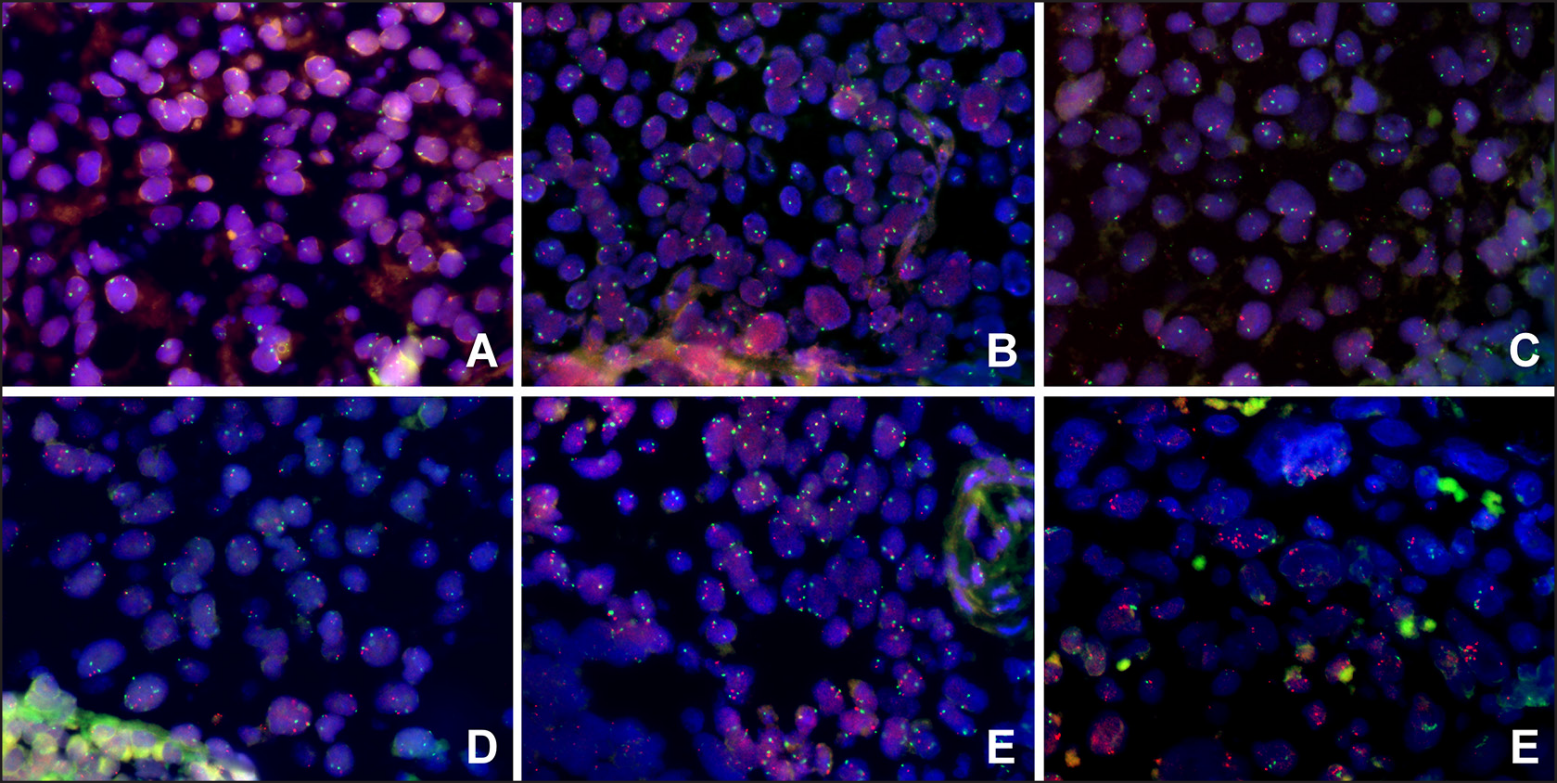
Evaluation of MET gene copy number status in patients with high MET expression in locoregionally advanced nasopharyngeal carcinoma using FISH **A–F.** Representative images of tumors with *MET* disomy **A.** low trisomy **B.** high trisomy **C.** low polysomy **D.** high polysomy **E.** and *MET* amplification **F.**

The *MET* copy number status of the patients with high MET expression (*n* = 139) was analyzed using fluorescent *in situ* hybridization (FISH). In total, 7/139 (5.0%) patients demonstrated *MET* amplification; 24 (17.3%), 29 (20.9%), 31 (22.3%), 29 (20.9%) and 19 (13.7%) patients displayed high polysomy, low polysomy, high trisomy, low trisomy and disomy, respectively (Figure 2A–2F). The average *MET* copy number per tumor cell ranged from 1.6 to 9.67 (mean, 3.23). The *MET/CEP7* ratio ranged from 0.9 to 4.1 (mean, 1.22).

The correlation between the *MET* amplification status and the clinicopathological characteristics of the patients with high MET expression are shown in Table 1. *MET* amplification was significantly associated with death (85.7% vs. 39.4%, *P* = 0.021). However, there was no significant association between the *MET* copy number status and any other clinicopathological factor.

### Correlation between *MET* copy number status and MET protein expression

In the 139 patients with high MET expression, the *MET* copy number status correlated significantly with MET protein expression (*r* = 0.629, *P* < 0.001, Spearman's rank; Table 2). The cases with a MET IHC score of 3+ had a markedly higher *MET* copy number compared to the cases with a MET IHC score of 2+. All seven tumors with *MET* amplification demonstrated a MET IHC score of 3+, while no *MET* amplification was detected in the 2+ tumors.

**Table 2:** Correlation between *MET* copy number status and MET protein expression in the 139 patients with high MET expression

	*MET* copy number status						
IHC Score	Disomy *n* (%)	Low Trisomy *n* (%)	High Trisomy *n* (%)	Low Polysomy *n* (%)	High Polysomy *n* (%)	Amplification *n* (%)	Total
2+	19 (100)	28 (96.6)	30 (96.8)	20 (69.0)	7 (29.2)	0 (0)	104
3+	0 (0)	1 (3.4)	1 (3.2)	9 (31.0)	17 (70.8)	7 (100)	35
**Total**	19	29	31	29	24	7	139

Abbreviation: IHC, immunohistochemistry score.

### Prognostic value of MET protein overexpression and *MET* amplification

**Figure 3:**
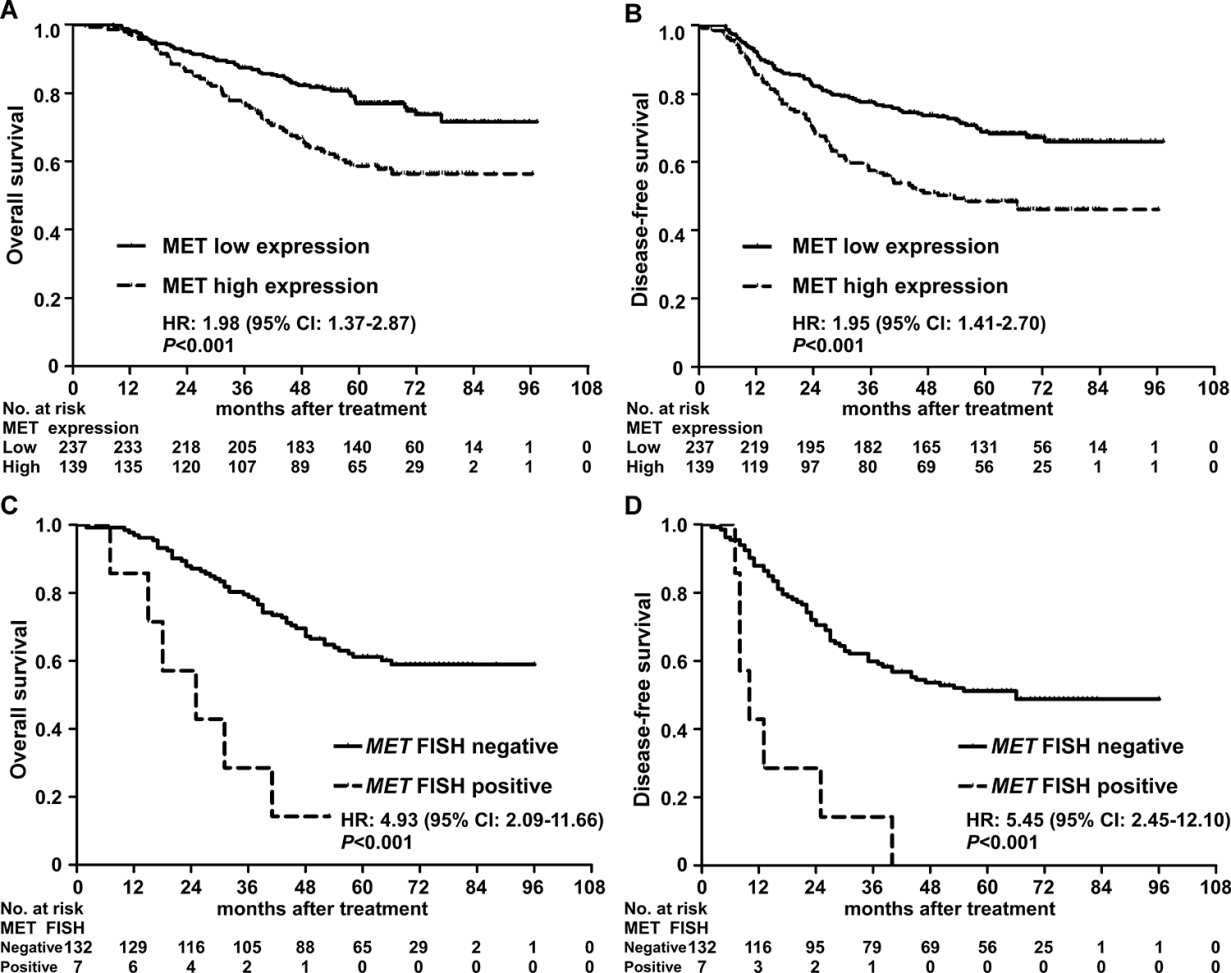
Kaplan-Meier overall survival and disease-free survival curves for patients with nasopharyngeal carcinoma stratified by MET protein expression and *MET* amplification status **A–B.** Overall survival and disease-free survival curves for patients stratified by MET protein expression. **C–D.** Overall survival and disease-free survival curves for patients with high MET expression stratified by *MET* amplification status. Hazard ratios (HRs) were calculated using the unadjusted Cox proportional hazards model; *P*-values were calculated using the unadjusted log-rank test. CI, confidence interval.

Univariate analysis indicated that MET protein expression, TNM stage, gender and WHO type had a significant impact on 5-year overall survival (OS) and disease-free survival (DFS; both *P* < 0.05; Table 3). The 5-year OS (58.6% vs. 76.9%, *P* < 0.001; Figure 3A) and DFS (48.5% vs. 68.8%, *P* < 0.001; Figure 3B) rates for patients with high MET expression were significantly lower than the corresponding rates for patients with low MET expression. In the group of patients with high MET expression, *MET* gene amplification was associated with poorer OS and DFS (*P* < 0.05) in univariate analysis, and patients with *MET* amplification had significantly poorer 5-year OS (48.5% vs. 68.8%, *P* < 0.001; Figure 3C) and DFS (0.0% vs. 51.1%, *P* < 0.001; Figure 3D).

**Table 3:** Univariate and multivariable Cox regression analysis of prognostic factors in 376 patients with locoregionally advanced NPC

Variable	Univariate analysis			Multivariate analysis		
	HR	95% CI	*P*-value	HR	95% CI	*P*-value
**Overall survival**						
MET expression (High vs. low)	1.98	1.37–2.87	**< 0.001**	1.99	1.38–2.87	**< 0.001**
TNM stage (IV vs. III)	1.91	1.32–2.77	**0.001**	1.93	1.33–2.79	**0.001**
Gender (Male vs. female)	2.05	1.23–3.44	**0.006**	1.99	1.19–3.33	**0.009**
Age (≥ 45 vs. < 45 years)	1.58	1.08–2.29	**0.02**	1.50	1.03–2.18	**0.035**
WHO type (IIb vs. IIa)	0.49	0.26–0.94	**0.03**			**NS**
VCA IgA (≥ 1:80 vs. < 1:80)	0.76	0.47–1.25	0.28			
EA IgA (≥ 1:10 vs. < 1:10)	0.82	0.53–1.27	0.37			
Chemotherapy (No vs. yes)	1.00	0.62–1.63	0.97			
Radiotherapy (2D-RT vs. IMRT)	1.06	0.58–1.92	0.86			
**Disease-free survival**						
MET expression (High vs. low)	1.95	1.41–2.70	**< 0.001**	1.85	1.33–2.57	**< 0.001**
TNM stage (IV vs. III)	1.69	1.22–2.33	**0.002**	1.71	1.24–2.36	**0.001**
Gender (Male vs. female)	1.55	1.03–2.32	**0.04**	1.52	1.02–2.29	**0.042**
WHO type (IIb vs. IIa)	0.42	0.24–0.75	**0.003**	0.54	0.30–0.97	**0.039**
Age (≥ 45 vs. < 45 years)	1.25	0.91–1.73	0.17			
VCA IgA (≥ 1:80 vs. < 1:80)	0.87	0.56–1.37	0.56			
EA IgA (≥ 1:10 vs. < 1:10)	0.96	0.64–1.42	0.82			
Chemotherapy (No vs. yes)	1.12	0.74–1.69	0.59			
Radiotherapy (2D-RT vs. IMRT)	1.13	0.65–1.97	0.66			

Abbreviations: WHO type IIa, differentiated non-keratinizing nasopharyngeal carcinoma; WHO type IIb, undifferentiated non-keratinizing nasopharyngeal carcinoma; VCA–IgA, viral capsid antigen immunoglobulin A; EA–IgA, early antigen immunoglobulin A; IMRT: intensity-modulated radiotherapy; 2D–RT: two-dimensional radiotherapy; HR, hazard ratio; NS, not significant.

Multivariate analysis revealed MET protein overexpression was an independent prognostic factor for OS (HR, 1.99; 95% CI, 1.38-2.87; *P* < 0.001) and DFS (HR, 1.85; 95% CI, 1.33-2.57; *P* < 0.001). TNM stage and gender were also found to be independent prognostic factors for OS and DFS (Table 3). Moreover, in the group of patients with high MET expression, *MET* amplification was also an independent prognostic factor for OS (HR, 4.24; 95% CI, 1.78-10.08; *P* < 0.001) and DFS (HR, 5.44; 95% CI, 2.44-12.09; *P* < 0.001). Taken together, these results indicate that MET protein overexpression is associated with unfavorable OS and DFS, and that *MET* amplification can be used to identify a subgroup of patients with high MET expression at risk of poor survival outcomes.

## DISCUSSION

In the present study, we report that MET protein was overexpressed in the tumors of 37.0% of the patients with locoregionally advanced NPC, with *MET* gene amplification observed in 5.0% of the patients with high MET expression. MET overexpression and *MET* amplification were both associated with a significantly poorer prognosis and were identified as independent prognostic factors for overall survival (OS) and disease-free survival (DFS). These results suggest that MET overexpression and *MET* amplification are potential biomarkers that could enable identification of a subgroup of patients with NPC who may be sensitive to MET-targeted therapy.

MET is aberrantly activated in a wide range of tumors, including lung cancer, gastric cancer, glioblastoma and renal-cell carcinoma [19, 22, 31, 32]. Aberrant MET signaling contributes to the initiation and progression of cancer [11, 12, 14, 16]. Accumulating evidence indicates that protein overexpression is one of the major mechanisms underlying the aberrant activation of MET, and overexpression of MET is associated with poor patient survival in a variety of types of cancer [25, 33–35]. Although several studies have evaluated the frequency and prognostic value of MET overexpression in NPC, the results of these studies are highly variable, with MET overexpression rates ranging from 51.5% to 91.1%, and some studies even reporting that MET had no significant prognostic value [28–30]. These controversial results may stem from the analysis of relatively small cohorts, as well as the use of different primary and secondary antibodies, staining protocols and scoring criteria. With the progression of MET inhibitors into clinical trials, an IHC assay was developed to evaluate MET protein expression using a rabbit monoclonal antibody SP44 [36]. During Phase II studies in gastric cancer (rilotumumab) and non-small cell lung cancer (onartuzumab), an IHC scoring algorithm based on the proportion of positive tumor cells and the staining intensity was established [36–39]. Using these IHC methods, antibody and scoring system, patients whose tumors overexpressed MET were demonstrated to benefit from MET-targeted therapies [36–39]. Using the same protocol, we identified that 37.0% of patients with locally advanced NPC overexpressed MET protein. Additionally, significant associations were observed between MET overexpression and locoregional failure, distant metastasis and death in patients with locoregionally advanced NPC, consistent with previous observations in gastric and lung cancer [25, 33–35]. Furthermore, overexpression of MET was associated with significantly poorer survival outcomes, suggesting that identification of MET overexpression using IHC may provide a potential biomarker to enable the individualized treatment of patients with NPC.

Gene amplification is another well-recognized mechanism that can lead to constitutive activation of MET [19, 40]. Based on the gold standard method of FISH, several studies have reported that *MET* is amplified in ~5% patients with non-small cell lung cancer and gastric cancer [19, 22, 34, 35, 41]. Additionally, patients with *MET* amplification have a poorer clinical prognosis [22, 42, 43]. Interestingly, MET protein overexpression correlated significantly with *MET* amplification, and *MET* amplification was rare in patients with low MET expression, ranging from 0% (0/283) to 0.6% (1/163) [34, 35]. Preliminary data from other studies have suggested that *MET* amplification can identify subgroups of patients with lung or gastric cancer who may benefit from MET inhibitors, suggesting that *MET* amplification as a may represent a putative biomarker [20, 27, 44, 45]. However, until now, the prevalence of *MET* amplification was unknown in NPC. This study demonstrated that 5.0% of patients with high MET expression had *MET* amplification. A significantly positive correlation was observed between *MET* amplification and MET protein overexpression, in agreement with previous data in gastric and lung cancer [35, 42]. All patients with *MET* amplification had a MET IHC score of 3+, and the majority of the remaining patients with a MET IHC score of 3+ had polysomy. Moreover, *MET* amplification was an independent prognostic factor for OS and DFS, further indicating that *MET* amplification may identify a subgroup of patients with high MET expression at risk of poorer survival who may benefit from MET inhibitor therapy.

In conclusion, this study evaluated the frequency of MET overexpression and *MET* amplification in locoregionally advanced NPC, and demonstrated that both MET overexpression and *MET* amplification were associated with poorer OS and DFS. These results enhance our knowledge of the mechanisms underlying aberrant activation of the MET pathway in NPC, and may help to establish MET as a novel prognostic biomarker and therapeutic target for the treatment of subgroups of patients with locoregionally advanced NPC.

## MATERIALS AND METHODS

### Clinical specimens

Paraffin-embedded biopsy specimens from 376 consecutive patients with histologically-confirmed, non-distant metastatic NPC treated at Sun Yat-sen University Cancer Center between January 2006 and December 2009 were evaluated; all samples were pathologically confirmed by two pathologists. No patients received radiotherapy or chemotherapy before biopsy. All MRI/CT materials and clinical records were reviewed and patients were restaged according to the 7^th^ edition of the AJCC Cancer Staging Manual. The clinical characteristics of the patients are summarized in Table 1. Written informed consent was obtained from all patients. This study was approved by the Institutional Ethical Review Board of Sun Yat-sen University Cancer Center.

### Patient treatment and follow-up

All patients received radiotherapy, as previously described [6]; 337 patients (89.6%) were treated with conventional two-dimensional radiotherapy (2D-RT) and 39 (10.4%) with intensity-modulated radiotherapy (IMRT). All patients had stage III (56.6%) or IV (43.4%) NPC. A total of 311 patients (82.7%) received concurrent platinum-based chemotherapy with or without neoadjuvant/adjuvant chemotherapy, as previously described [4, 5]. Concurrent chemotherapy consisted of cisplatin administered on weeks 1, 4 and 7 of radiotherapy, or weekly. Neoadjuvant and adjuvant chemotherapy consisted of cisplatin with 5-flurouracil or taxanes every three weeks for three cycles.

### Follow-up

All patients were followed-up at least every 3 months during the first 2 years and then every 6 months thereafter until death. Median follow-up was 62.4 months (range, 2.6–97.3 months). The following end points were assessed: overall survival (OS) and disease-free survival (DFS). OS was calculated from the first day of treatment to death; DFS, from the first day of treatment to disease progression or death from any cause.

### Immunohistochemistry

Immunohistochemistry (IHC) for MET was performed using an automatic staining system (Bench Mark ULTRA; Ventana Medical Systems, Tucson, AZ, USA), according to the manufacturer's instructions with CONFIRM anti-total MET antibody (SP44; rabbit monoclonal primary antibody; Ventana Medical Systems). Immunostaining was evaluated on the basis of the staining intensity (negative, weak, moderate, strong) and the percentage of cells stained, according to the MET IHC scoring criteria for non-small cell lung cancer [36, 37]. The MET IHC scores ranged from 0 to 3+ as follows: 0 (no staining or < 50% of tumor cells with any intensity); 1+ (≥ 50% of tumor cells with weak or higher staining intensity and < 50% with moderate or higher intensity); 2+ (≥ 50% of tumor cells with moderate or higher staining intensity and < 50% strong intensity); 3+ (≥ 50% of tumor cells with strong staining intensity). Scores of 2+ and 3+ were defined as high MET expression, scores of 1+ and 0 were defined as low MET expression. The IHC scores were independently evaluated by two pathologists who were blinded to the clinical and molecular characteristics of the patients.

### Fluorescence *in situ* hybridization

*MET* copy number was evaluated using fluorescence *in situ* hybridization (FISH), as previously described [46]. Briefly, 4 μm tissue sections were hybridized overnight with the Vysis MET Spectrum Red FISH Probe (Abbott Molecular, Chicago, IL, USA) and control Vysis CEP7 centromere Spectrum Green Probe (Abbott Molecular). The entire area of each section was scanned using a 100× objective (Olympus, Tokyo, Japan) and appropriate filter sets (Vysis; Abbott Molecular). The *MET* (red) and *CEP7* (green) signals were evaluated for at least 100 non-overlapping nuclei in each slide.

Patients were classified into six groups based on the *MET* copy number status, according to the University of Colorado Cancer Center criteria [46]: 1, disomy (< 90% of tumor cells containing ≤ 2 MET signals); 2, low trisomy (≥ 40% of tumor cells containing ≤ 2 MET signals, 10–40% of tumor cells containing three MET signals, and < 10% of tumor cells containing ≥ 4 MET signals); 3, high trisomy (≥ 40% of tumor cells containing ≤ 2 MET signals, and ≥ 40% of tumor cells containing three MET signals, and < 10% of tumor cells containing ≥ 4 MET signals); 4, low polysomy (10–40% of tumor cells containing ≥ 4 MET signals); 5, high polysomy (≥ 40% of tumor cells containing ≥ 4 MET signals) and 6, *MET* amplification (MET/CEP7 ratio ≥ 2.0 or ≥ 10% of tumor cells containing ≥ 15 MET signals). *MET* amplification was defined as *MET* FISH-positive; the remaining cases were classified as *MET* FISH-negative. *MET* copy number status was determined by two investigators while blinded to the clinical and molecular characteristics of the patients and IHC data.

### Statistical analysis

All statistical analysis was performed using SPSS 16.0 software (IBM, Armonk, NY, USA). Categorical variables were compared using the χ^2^ and Fisher's exact tests. The association between *MET* FISH status and MET protein expression was evaluated using the Spearman's rank method. Actuarial rates were calculated using the Kaplan-Meier method; differences were compared using the log-rank test. Multivariate analysis using a Cox proportional hazards model was performed to calculate hazard ratios (HRs) and 95% confidence intervals (CIs), and assess independent significance by backward elimination of insignificant explanatory variables. Covariates including host factors (i.e., age and gender), tumor factors (i.e., WHO type, clinical stage, T and N classification), radiotherapy (RT) technique and chemotherapeutic intervention (i.e., RT alone or chemo-RT) were included in all tests. All tests were two-tailed; *P*-values < 0.05 were considered statistically significant.
